# Chlorido[6-phenyl-4-(*p*-tol­yl)-2,2′-bipyridyl-κ^2^ *N*,*N*′]platinum(II)

**DOI:** 10.1107/S1600536808020473

**Published:** 2008-07-09

**Authors:** Hong Bo Lu, Guo Qiang Lv, Jin Lin Zuo, Jia Xiang Yang

**Affiliations:** a Key Laboratory of Special Display Technology and Academy of Opto-Electronic Technology, Hefei University of Technology, Ministry of Education, Hefei 230009, People’s Republic of China; b Deparment of Chemistry, Anhui University, Hefei 230039, People’s Republic of China

## Abstract

The asymmetric unit of the title compound, [Pt(C_23_H_17_N_2_)Cl], contains two independent mol­ecules with distinct dihedral angles between the central pyridyl and methylbenzene rings [7.77 (2) and 24.07 (2)°]. Short inter­molecular distances [3.582 (6) and 3.600 (6) Å] between the outer pyridine and the PtNC_3_ and PtN_2_C_2_ rings, respectively, indicate the existence of π–π inter­actions, which link the mol­ecules into stacks along the *a* axis. The crystal structure is further stabilized by weak C—H⋯π inter­actions.

## Related literature

For related literature, see: Allen *et al.* (1987 ▶); Catalano *et al.* (2000 ▶); Kubicki *et al.* (2002 ▶).
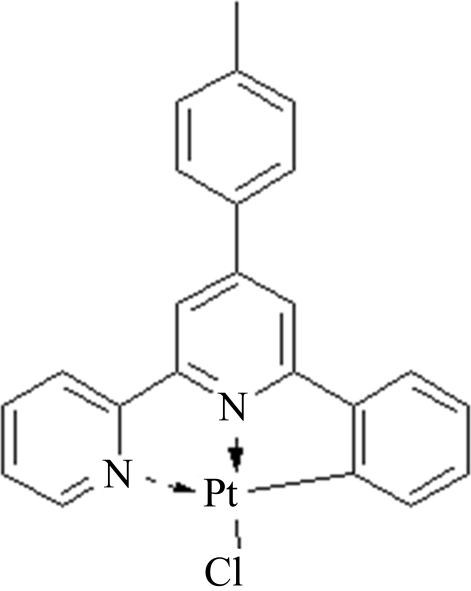


## Experimental

### 

#### Crystal data

[Pt(C_23_H_17_N_2_)Cl]
*M* *_r_* = 551.92
Monoclinic, 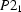

*a* = 7.379 (5) Å
*b* = 18.066 (5) Å
*c* = 14.222 (5) Å
β = 102.551 (5)°
*V* = 1850.6 (15) Å^3^
*Z* = 4
Mo *K*α radiation
μ = 7.74 mm^−1^
*T* = 298 (2) K
0.50 × 0.30 × 0.20 mm

#### Data collection

Bruker APEX area-dectector diffractometer
Absorption correction: multi-scan (*SADABS*; Bruker, 2002 ▶) *T*_min_ = 0.113, *T*_max_ = 0.307 (expected range = 0.078–0.213)
14983 measured reflections
8028 independent reflections
6615 reflections with *I* > 2σ(*I*)
*R*_int_ = 0.035

#### Refinement

*R*[*F* ^2^ > 2σ(*F* ^2^)] = 0.038
*wR*(*F* ^2^) = 0.110
*S* = 0.72
8028 reflections
489 parameters
1 restraint
H-atom parameters constrained
Δρ_max_ = 1.91 e Å^−3^
Δρ_min_ = −0.65 e Å^−3^
Absolute structure: Flack (1983 ▶), with 3598 Friedel pairs
Flack parameter: 0.001 (11)

### 

Data collection: *SMART* (Bruker, 2002 ▶); cell refinement: *SAINT* (Bruker, 2002 ▶); data reduction: *SAINT*; program(s) used to solve structure: *SHELXS97* (Sheldrick, 2008 ▶); program(s) used to refine structure: *SHELXL97* (Sheldrick, 2008 ▶); molecular graphics: *ORTEPII* (Johnson, 1976 ▶); software used to prepare material for publication: *SHELXL97*.

## Supplementary Material

Crystal structure: contains datablocks I, global. DOI: 10.1107/S1600536808020473/cv2424sup1.cif

Structure factors: contains datablocks I. DOI: 10.1107/S1600536808020473/cv2424Isup2.hkl

Additional supplementary materials:  crystallographic information; 3D view; checkCIF report

## Figures and Tables

**Table 1:** Selected interatomic distances (Å) *Cg*1, *Cg*2 and *Cg*3 are the centroids of the N4/C24–C28, Pt1/N1/C10/C11/C16 and Pt1/N1/N2/C5/C6 rings, respectively.

N3—Pt2	1.941 (8)
Pt2—C39	2.001 (11)
Pt2—N4	2.130 (9)
Pt2—Cl2	2.302 (3)
Pt1—N1	1.932 (7)
Pt1—C16	1.981 (9)
Pt1—N2	2.116 (8)
Pt1—Cl1	2.299 (3)
*Cg*1⋯*Cg*2^i^	3.582 (6)
*Cg*1⋯*Cg*3	3.600 (6)

Symmetry codes: (i) 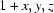.

**Table 2:** Hydrogen-bond geometry (Å, °) *Cg*4 and *Cg*5 are the centroids of the Pt2/N3/C33/C34/C39 and C41–C45 rings, respectively.

*D*—H⋯*A*	*D*—H	H⋯*A*	*D*⋯*A*	*D*—H⋯*A*
C3—H3⋯*Cg*4^ii^	0.93	2.87	3.650 (14)	142
C14—H14⋯*Cg*5^iii^	0.93	2.71	3.445 (14)	136

Symmetry codes: (ii 
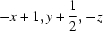
; (iii) 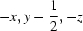.

**Fig. 1.**
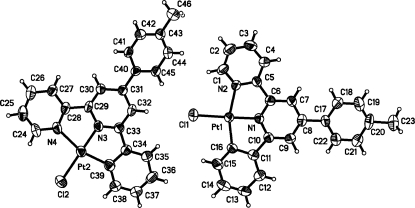
: The content of asymmetric unit of the title compound showing the atomic numbering and 50% probability displacement ellipsoids.

## supplementary crystallographic information

### Comment

Recently, the bonding interaction between the closed-shell metal atoms or ions is gaining increasing attention, and while there exist numerous examples of fluorophenyl and cyanate platinum(II) centers aggregating with s^2^ ions such as Tl(I) or Pb(II) and d^10^ ions such as Au(I) or Ag(I)(Catalano *et al.*, 2000), there are few reports of similar association in the case of alkyl platinum(II) complexes. As a part of our ongoing investigation on platinum complexes, the title compound (I) has been prepared and its crystal structure is presented here.

There are two crystallographically independent molecules in the asymmetric unit of (I) (Fig. 1). Each molecule contains a Pt atom coordinated in a distorted square-planar configuration with two Pt-N, one Pt-C and one Pt-Cl bonds (Table 1). Bond lengths and angles in the two molecules are similar and in a argreement with the values reported in the literature (Allen *et al.*, 1987). The dihedral angles formed by the C17—C22 and C40—C45 benzene rings with N1/C6—C10 and N3/C29—C33 are 24.07 (2)°, 7.77 (2)°, respectively. The crystal packing of the structure exhibits π-π interactions proved by short intermolecular *Cg*1···*Cg*2 and *Cg*1···*Cg*3 distances of 3.582 (6) and 3.600 (6) Å, respectively; *Cg*1, *Cg*2 and *Cg*3 are centroids of N4/C24—C28, Pt1/N1/C10/C11/C16 and Pt1/N1/N2/C5/C6 rings, respectively (Table 1), which link the molecules into stacks along *a* axis. The crystal structure is further stabilized by the weak C—H···π interactions (Kubicki *et al.*, 2002; Table 2).

### Experimental

For the preparation of 3,6-diimidazolyl-9-ethylcarbazole, A mixture of 4-(*p*-tolyl)-6-phenyl-2,2'-bipyridine (96.72 mg, 0.30 mmol) and K_2_PtCl_4_ (124.58 mg, 0.30 mmol) were heated at 363 K with CH_3_CN (10 ml) as solvent for 18 h. The mixture was cooled to room temperature. Then it was filtered and concentrated, the re-crystallization from DMF produced red single crystals (38.90 mg, Yield 83.88%).

### Refinement

All hydrogen atoms were placed in geometrically idealized positions and constrained to ride on their parent atoms, with C—H = 0.93 - 0.96 Å and *U*_iso_(H) = 1.2 or 1.5 *U*_eq_(C). The highest residual peak [1.91 e Å^-3^] is situated 0.11 Å at atom H30.

### Crystal data

**Table:**

[Pt(C_23_H_17_N_2_)Cl]	*Z* = 4
*M**_r_* = 551.92	*F*_000_ = 1056
Monoclinic, *P*2_1_	*D*_x_ = 1.981 Mg m^−^^3^
*a* = 7.379 (5) Å	Mo *K*α radiation λ = 0.71069 Å
*b* = 18.066 (5) Å	µ = 7.74 mm^−^^1^
*c* = 14.222 (5) Å	*T* = 298 (2) K
β = 102.551 (5)º	Needle, red
*V* = 1850.6 (15) Å^3^	0.50 × 0.30 × 0.20 mm

### Data collection

**Table:**

Bruker APEX area-dectector diffractometer	8028 independent reflections
Radiation source: fine-focus sealed tube	6615 reflections with *I* > 2σ(*I*)
Monochromator: graphite	*R*_int_ = 0.035
*T* = 298(2) K	θ_max_ = 27.5º
φ and ω scans	θ_min_ = 1.9º
Absorption correction: multi-scan(SADABS; Bruker, 2002)	*h* = −9→9
*T*_min_ = 0.113, *T*_max_ = 0.307	*k* = −23→23
14983 measured reflections	*l* = −18→18

### Refinement

**Table:**

Refinement on *F*^2^	Hydrogen site location: inferred from neighbouring sites
Least-squares matrix: full	H-atom parameters constrained
*R*[*F*^2^ > 2σ(*F*^2^)] = 0.038	*w* = 1/[σ^2^(*F*_o_^2^) + (0.1*P*)^2^] where *P* = (*F*_o_^2^ + 2*F*_c_^2^)/3
*wR*(*F*^2^) = 0.110	(Δ/σ)_max_ = 0.001
*S* = 0.72	Δρ_max_ = 1.91 e Å^−^^3^
8028 reflections	Δρ_min_ = −0.65 e Å^−^^3^
489 parameters	Extinction correction: none
1 restraint	Absolute structure: Flack (1983), 3598 Friedel pairs
Primary atom site location: structure-invariant direct methods	Flack parameter: 0.001 (11)
Secondary atom site location: difference Fourier map	

### Special details

**Table:**

Geometry. All e.s.d.'s (except the e.s.d. in the dihedral angle between two l.s. planes) are estimated using the full covariance matrix. The cell e.s.d.'s are taken into account individually in the estimation of e.s.d.'s in distances, angles and torsion angles; correlations between e.s.d.'s in cell parameters are only used when they are defined by crystal symmetry. An approximate (isotropic) treatment of cell e.s.d.'s is used for estimating e.s.d.'s involving l.s. planes.
Refinement. Refinement of *F*^2^ against ALL reflections. The weighted *R*-factor *wR* and goodness of fit *S* are based on *F*^2^, conventional *R*-factors *R* are based on *F*, with *F* set to zero for negative *F*^2^. The threshold expression of *F*^2^ > σ(*F*^2^) is used only for calculating *R*-factors(gt) *etc*. and is not relevant to the choice of reflections for refinement. *R*-factors based on *F*^2^ are statistically about twice as large as those based on *F*, and *R*- factors based on ALL data will be even larger.

### Fractional atomic coordinates and isotropic or equivalent isotropic displacement parameters (Å^2^)

**Table:**

	*x*	*y*	*z*	*U*_iso_*/*U*_eq_	
C19	0.7020 (18)	0.2281 (9)	0.3338 (8)	0.071 (3)	
H19	0.7383	0.2734	0.3628	0.085*	
N3	0.0093 (11)	0.0772 (4)	0.1479 (6)	0.0455 (18)	
Pt2	−0.09764 (5)	−0.01160 (2)	0.08126 (2)	0.04713 (11)	
Pt1	0.35781 (5)	0.143731 (18)	−0.25831 (2)	0.04136 (10)	
Cl2	−0.2212 (5)	−0.11757 (18)	0.0028 (2)	0.0741 (8)	
Cl1	0.2643 (5)	0.1428 (2)	−0.42342 (17)	0.0687 (7)	
N1	0.4326 (9)	0.1481 (5)	−0.1195 (5)	0.0391 (14)	
C44	0.2848 (18)	0.3505 (7)	0.4362 (7)	0.061 (3)	
H44	0.2739	0.3580	0.4994	0.074*	
C39	−0.0498 (14)	−0.0514 (6)	0.2157 (7)	0.052 (2)	
C8	0.5388 (12)	0.1543 (6)	0.0788 (6)	0.044 (2)	
C27	−0.0172 (14)	0.1830 (6)	−0.0774 (7)	0.048 (2)	
H27	0.0347	0.2287	−0.0569	0.057*	
C17	0.5989 (13)	0.1582 (5)	0.1879 (6)	0.044 (2)	
C40	0.2257 (12)	0.2733 (5)	0.2945 (6)	0.0400 (19)	
C33	0.0607 (13)	0.0765 (5)	0.2432 (7)	0.044 (2)	
C9	0.4583 (13)	0.0905 (5)	0.0326 (7)	0.045 (2)	
H9	0.4410	0.0493	0.0689	0.054*	
C45	0.2185 (16)	0.2857 (6)	0.3890 (7)	0.050 (2)	
H45	0.1678	0.2496	0.4222	0.060*	
C31	0.1507 (13)	0.2049 (5)	0.2432 (6)	0.041 (2)	
C41	0.3111 (14)	0.3287 (6)	0.2493 (6)	0.047 (2)	
H41	0.3226	0.3217	0.1861	0.056*	
C12	0.2565 (16)	−0.0365 (6)	−0.0924 (9)	0.058 (3)	
H12	0.2838	−0.0446	−0.0262	0.069*	
C10	0.4033 (12)	0.0878 (5)	−0.0677 (7)	0.0404 (19)	
C42	0.3768 (16)	0.3918 (6)	0.2959 (8)	0.056 (3)	
H42	0.4299	0.4275	0.2633	0.067*	
C43	0.3675 (18)	0.4046 (6)	0.3900 (8)	0.061 (3)	
N4	−0.1077 (11)	0.0607 (5)	−0.0380 (6)	0.0500 (19)	
C32	0.1347 (12)	0.1397 (6)	0.2932 (6)	0.0457 (19)	
H32	0.1736	0.1384	0.3599	0.055*	
C29	0.0292 (12)	0.1399 (7)	0.0952 (5)	0.0416 (18)	
C25	−0.1501 (16)	0.0992 (8)	−0.2003 (8)	0.064 (3)	
H25	−0.1893	0.0876	−0.2653	0.077*	
N2	0.4760 (11)	0.2509 (5)	−0.2403 (5)	0.0440 (17)	
C16	0.2736 (12)	0.0437 (5)	−0.2302 (7)	0.0411 (19)	
C21	0.637 (2)	0.1009 (9)	0.3453 (9)	0.081 (4)	
H21	0.6283	0.0592	0.3823	0.097*	
C37	−0.0303 (18)	−0.1362 (7)	0.3487 (9)	0.066 (3)	
H37	−0.0465	−0.1839	0.3699	0.079*	
C38	−0.0821 (19)	−0.1205 (7)	0.2512 (8)	0.064 (3)	
H38	−0.1395	−0.1570	0.2089	0.077*	
C34	0.0295 (14)	0.0036 (5)	0.2848 (7)	0.046 (2)	
C30	0.0987 (12)	0.2031 (5)	0.1427 (6)	0.0403 (19)	
H30	0.1115	0.2455	0.1076	0.048*	
C46	0.436 (2)	0.4749 (8)	0.4407 (10)	0.088 (4)	
H46A	0.4440	0.5125	0.3941	0.131*	
H46B	0.5562	0.4669	0.4814	0.131*	
H46C	0.3510	0.4905	0.4793	0.131*	
C11	0.3080 (12)	0.0276 (6)	−0.1299 (7)	0.044 (2)	
C6	0.5122 (13)	0.2106 (5)	−0.0761 (7)	0.044 (2)	
C18	0.6581 (17)	0.2241 (7)	0.2346 (8)	0.059 (3)	
H18	0.6682	0.2663	0.1985	0.070*	
C28	−0.0335 (13)	0.1287 (5)	−0.0092 (6)	0.045 (2)	
C22	0.594 (2)	0.0974 (8)	0.2454 (8)	0.074 (4)	
H22	0.5608	0.0519	0.2161	0.089*	
C7	0.5620 (15)	0.2149 (6)	0.0231 (7)	0.047 (2)	
H7	0.6112	0.2587	0.0526	0.057*	
C24	−0.1648 (14)	0.0476 (7)	−0.1329 (7)	0.056 (3)	
H24	−0.2161	0.0018	−0.1530	0.068*	
C13	0.1620 (16)	−0.0893 (6)	−0.1555 (9)	0.062 (3)	
H13	0.1259	−0.1337	−0.1320	0.074*	
C4	0.6150 (16)	0.3362 (6)	−0.1195 (8)	0.057 (2)	
H4	0.6526	0.3487	−0.0548	0.068*	
C35	0.0742 (16)	−0.0126 (7)	0.3828 (8)	0.060 (2)	
H35	0.1243	0.0238	0.4271	0.072*	
C5	0.5346 (14)	0.2690 (5)	−0.1462 (7)	0.045 (2)	
C14	0.1223 (16)	−0.0757 (7)	−0.2530 (10)	0.062 (3)	
H14	0.0578	−0.1111	−0.2948	0.075*	
C15	0.1765 (14)	−0.0102 (8)	−0.2903 (8)	0.056 (2)	
H15	0.1472	−0.0024	−0.3566	0.067*	
C2	0.5799 (19)	0.3664 (7)	−0.2857 (9)	0.068 (3)	
H2	0.5929	0.3992	−0.3342	0.082*	
C1	0.5009 (15)	0.2984 (7)	−0.3080 (8)	0.057 (3)	
H1	0.4633	0.2850	−0.3724	0.069*	
C26	−0.0770 (16)	0.1693 (7)	−0.1738 (7)	0.063 (3)	
H26	−0.0694	0.2052	−0.2197	0.075*	
C3	0.6396 (17)	0.3855 (7)	−0.1910 (9)	0.065 (3)	
H3	0.6962	0.4311	−0.1744	0.078*	
C20	0.6946 (17)	0.1699 (9)	0.3895 (7)	0.071 (4)	
C36	0.044 (2)	−0.0835 (7)	0.4141 (9)	0.069 (3)	
H36	0.0738	−0.0948	0.4794	0.082*	
C23	0.744 (2)	0.1739 (10)	0.5006 (9)	0.086 (4)	
H23A	0.8454	0.1408	0.5249	0.129*	
H23B	0.6383	0.1597	0.5254	0.129*	
H23C	0.7798	0.2235	0.5205	0.129*	

### Atomic displacement parameters (Å^2^)

**Table:**

	*U*^11^	*U*^22^	*U*^33^	*U*^12^	*U*^13^	*U*^23^
C19	0.077 (8)	0.085 (9)	0.050 (6)	−0.004 (7)	0.013 (6)	−0.018 (6)
N3	0.051 (4)	0.042 (4)	0.038 (4)	0.008 (4)	−0.003 (3)	−0.003 (3)
Pt2	0.0528 (2)	0.04704 (19)	0.04067 (19)	0.00263 (18)	0.00828 (15)	−0.00699 (16)
Pt1	0.04689 (18)	0.03779 (16)	0.03740 (17)	0.00176 (16)	0.00475 (13)	0.00092 (15)
Cl2	0.102 (2)	0.0613 (17)	0.0567 (16)	−0.0187 (17)	0.0123 (16)	−0.0182 (14)
Cl1	0.0953 (19)	0.0648 (14)	0.0393 (11)	−0.0045 (19)	−0.0002 (12)	0.0015 (14)
N1	0.045 (3)	0.035 (3)	0.037 (3)	0.009 (4)	0.008 (3)	−0.003 (4)
C44	0.084 (8)	0.059 (7)	0.037 (5)	−0.006 (6)	0.005 (5)	−0.004 (5)
C39	0.056 (6)	0.055 (6)	0.048 (5)	0.000 (5)	0.016 (5)	−0.009 (5)
C8	0.039 (4)	0.055 (6)	0.038 (4)	0.015 (4)	0.010 (3)	0.000 (4)
C27	0.055 (6)	0.047 (5)	0.039 (5)	0.012 (5)	0.009 (4)	0.003 (4)
C17	0.048 (5)	0.049 (6)	0.035 (4)	0.008 (4)	0.008 (3)	−0.005 (4)
C40	0.048 (5)	0.033 (4)	0.039 (4)	0.007 (4)	0.010 (4)	0.005 (4)
C33	0.044 (5)	0.041 (5)	0.043 (5)	0.005 (4)	0.005 (4)	0.002 (4)
C9	0.046 (5)	0.040 (5)	0.049 (5)	0.001 (4)	0.012 (4)	0.004 (4)
C45	0.073 (6)	0.040 (5)	0.035 (5)	0.010 (5)	0.007 (4)	0.003 (4)
C31	0.049 (5)	0.038 (5)	0.036 (4)	0.006 (4)	0.010 (4)	−0.001 (4)
C41	0.067 (6)	0.044 (5)	0.031 (4)	0.007 (5)	0.014 (4)	0.003 (4)
C12	0.068 (7)	0.049 (6)	0.056 (6)	0.001 (5)	0.012 (5)	0.010 (5)
C10	0.041 (5)	0.037 (4)	0.042 (5)	0.008 (4)	0.006 (4)	−0.003 (4)
C42	0.067 (6)	0.045 (5)	0.053 (6)	0.005 (5)	0.005 (5)	0.001 (5)
C43	0.080 (8)	0.047 (5)	0.044 (6)	0.003 (6)	−0.011 (5)	0.003 (5)
N4	0.044 (4)	0.058 (5)	0.041 (4)	0.001 (4)	−0.005 (3)	−0.006 (4)
C32	0.052 (5)	0.055 (5)	0.030 (4)	0.008 (5)	0.008 (3)	0.003 (5)
C29	0.048 (4)	0.051 (5)	0.027 (3)	0.010 (5)	0.009 (3)	0.005 (4)
C25	0.067 (7)	0.091 (9)	0.035 (5)	0.014 (7)	0.012 (5)	−0.010 (6)
N2	0.049 (4)	0.046 (4)	0.035 (4)	−0.005 (4)	0.003 (3)	0.005 (3)
C16	0.040 (4)	0.035 (4)	0.045 (5)	0.004 (4)	0.003 (4)	0.000 (4)
C21	0.119 (12)	0.073 (9)	0.048 (7)	−0.011 (8)	0.015 (7)	0.010 (6)
C37	0.082 (8)	0.055 (6)	0.060 (7)	−0.013 (6)	0.015 (6)	0.007 (5)
C38	0.077 (7)	0.053 (6)	0.056 (7)	−0.010 (6)	0.002 (6)	0.006 (5)
C34	0.056 (5)	0.035 (5)	0.047 (5)	0.010 (4)	0.015 (4)	0.000 (4)
C30	0.042 (5)	0.041 (5)	0.037 (4)	0.005 (4)	0.007 (4)	−0.001 (4)
C46	0.127 (12)	0.060 (8)	0.070 (8)	−0.016 (8)	0.010 (8)	−0.007 (6)
C11	0.033 (4)	0.044 (5)	0.052 (5)	0.002 (4)	0.005 (4)	−0.001 (4)
C6	0.041 (5)	0.043 (5)	0.045 (5)	0.000 (4)	0.006 (4)	0.000 (4)
C18	0.067 (7)	0.060 (6)	0.050 (6)	−0.005 (6)	0.015 (5)	0.007 (5)
C28	0.054 (5)	0.051 (6)	0.031 (4)	0.020 (4)	0.011 (4)	−0.002 (4)
C22	0.108 (10)	0.066 (8)	0.044 (6)	−0.007 (8)	0.007 (6)	−0.003 (5)
C7	0.060 (6)	0.041 (5)	0.041 (5)	0.001 (4)	0.010 (4)	−0.006 (4)
C24	0.050 (5)	0.072 (7)	0.042 (5)	−0.002 (5)	0.000 (4)	−0.016 (5)
C13	0.063 (7)	0.041 (5)	0.078 (8)	−0.009 (5)	0.005 (6)	−0.002 (5)
C4	0.076 (7)	0.041 (5)	0.053 (6)	0.000 (5)	0.014 (5)	0.001 (5)
C35	0.077 (7)	0.045 (5)	0.054 (5)	0.001 (6)	0.006 (5)	0.005 (5)
C5	0.052 (5)	0.042 (5)	0.040 (5)	0.002 (4)	0.008 (4)	−0.004 (4)
C14	0.058 (6)	0.047 (6)	0.082 (9)	−0.008 (5)	0.013 (6)	−0.016 (6)
C15	0.053 (5)	0.056 (6)	0.053 (5)	−0.010 (6)	0.000 (4)	−0.006 (6)
C2	0.084 (8)	0.060 (7)	0.064 (7)	−0.003 (7)	0.025 (6)	0.018 (6)
C1	0.061 (6)	0.055 (6)	0.054 (6)	−0.005 (5)	0.011 (5)	0.019 (5)
C26	0.066 (7)	0.081 (8)	0.039 (5)	0.017 (6)	0.006 (5)	0.003 (5)
C3	0.069 (7)	0.048 (6)	0.076 (8)	−0.017 (6)	0.011 (6)	−0.005 (6)
C20	0.069 (7)	0.111 (11)	0.030 (5)	0.006 (7)	0.007 (5)	0.006 (6)
C36	0.093 (8)	0.063 (7)	0.055 (7)	0.001 (7)	0.026 (6)	0.017 (6)
C23	0.105 (10)	0.105 (11)	0.048 (7)	0.000 (9)	0.016 (7)	−0.010 (7)

### Geometric parameters (Å, °)

**Table:**

C19—C20	1.32 (2)	C32—H32	0.9300
C19—C18	1.379 (16)	C29—C30	1.369 (14)
C19—H19	0.9300	C29—C28	1.470 (11)
N3—C33	1.325 (12)	C25—C24	1.358 (18)
N3—C29	1.384 (14)	C25—C26	1.397 (18)
N3—Pt2	1.941 (8)	C25—H25	0.9300
Pt2—C39	2.001 (11)	N2—C1	1.333 (13)
Pt2—N4	2.130 (9)	N2—C5	1.354 (12)
Pt2—Cl2	2.302 (3)	C16—C15	1.388 (14)
Pt1—N1	1.932 (7)	C16—C11	1.422 (14)
Pt1—C16	1.981 (9)	C21—C22	1.388 (17)
Pt1—N2	2.116 (8)	C21—C20	1.42 (2)
Pt1—Cl1	2.299 (3)	C21—H21	0.9300
N1—C6	1.357 (13)	C37—C36	1.360 (18)
N1—C10	1.358 (13)	C37—C38	1.384 (16)
C44—C45	1.385 (15)	C37—H37	0.9300
C44—C43	1.390 (17)	C38—H38	0.9300
C44—H44	0.9300	C34—C35	1.391 (14)
C39—C38	1.387 (17)	C30—H30	0.9300
C39—C34	1.432 (14)	C46—H46A	0.9600
C8—C7	1.384 (14)	C46—H46B	0.9600
C8—C9	1.394 (14)	C46—H46C	0.9600
C8—C17	1.520 (12)	C6—C7	1.381 (13)
C27—C26	1.368 (14)	C6—C5	1.486 (14)
C27—C28	1.403 (14)	C18—H18	0.9300
C27—H27	0.9300	C22—H22	0.9300
C17—C22	1.374 (16)	C7—H7	0.9300
C17—C18	1.388 (16)	C24—H24	0.9300
C40—C45	1.375 (13)	C13—C14	1.375 (17)
C40—C41	1.410 (13)	C13—H13	0.9300
C40—C31	1.480 (13)	C4—C5	1.368 (14)
C33—C32	1.393 (15)	C4—C3	1.393 (16)
C33—C34	1.482 (13)	C4—H4	0.9300
C9—C10	1.396 (13)	C35—C36	1.390 (17)
C9—H9	0.9300	C35—H35	0.9300
C45—H45	0.9300	C14—C15	1.391 (18)
C31—C32	1.395 (14)	C14—H14	0.9300
C31—C30	1.397 (13)	C15—H15	0.9300
C41—C42	1.354 (15)	C2—C3	1.367 (19)
C41—H41	0.9300	C2—C1	1.367 (18)
C12—C11	1.364 (15)	C2—H2	0.9300
C12—C13	1.390 (16)	C1—H1	0.9300
C12—H12	0.9300	C26—H26	0.9300
C10—C11	1.480 (14)	C3—H3	0.9300
C42—C43	1.374 (16)	C20—C23	1.544 (15)
C42—H42	0.9300	C36—H36	0.9300
C43—C46	1.493 (17)	C23—H23A	0.9600
N4—C24	1.344 (13)	C23—H23B	0.9600
N4—C28	1.370 (13)	C23—H23C	0.9600
			
Cg1···Cg2^i^	3.582 (6)	Cg1···Cg3	3.600 (6)
			
C20—C19—C18	122.7 (13)	C22—C21—C20	118.3 (12)
C20—C19—H19	118.7	C22—C21—H21	120.9
C18—C19—H19	118.7	C20—C21—H21	120.9
C33—N3—C29	121.4 (8)	C36—C37—C38	121.6 (11)
C33—N3—Pt2	119.2 (7)	C36—C37—H37	119.2
C29—N3—Pt2	119.4 (6)	C38—C37—H37	119.2
N3—Pt2—C39	82.1 (4)	C37—C38—C39	121.1 (11)
N3—Pt2—N4	79.8 (3)	C37—C38—H38	119.4
C39—Pt2—N4	161.8 (4)	C39—C38—H38	119.4
N3—Pt2—Cl2	179.3 (2)	C35—C34—C39	121.0 (9)
C39—Pt2—Cl2	97.7 (3)	C35—C34—C33	124.3 (9)
N4—Pt2—Cl2	100.5 (2)	C39—C34—C33	114.7 (9)
N1—Pt1—C16	82.2 (4)	C29—C30—C31	121.0 (9)
N1—Pt1—N2	79.6 (3)	C29—C30—H30	119.5
C16—Pt1—N2	161.8 (3)	C31—C30—H30	119.5
N1—Pt1—Cl1	177.9 (3)	C43—C46—H46A	109.5
C16—Pt1—Cl1	99.3 (3)	C43—C46—H46B	109.5
N2—Pt1—Cl1	98.9 (2)	H46A—C46—H46B	109.5
C6—N1—C10	121.6 (7)	C43—C46—H46C	109.5
C6—N1—Pt1	119.9 (6)	H46A—C46—H46C	109.5
C10—N1—Pt1	118.5 (6)	H46B—C46—H46C	109.5
C45—C44—C43	120.7 (10)	C12—C11—C16	124.1 (9)
C45—C44—H44	119.7	C12—C11—C10	121.8 (9)
C43—C44—H44	119.7	C16—C11—C10	114.1 (8)
C38—C39—C34	116.9 (10)	N1—C6—C7	120.4 (9)
C38—C39—Pt2	131.6 (8)	N1—C6—C5	112.7 (8)
C34—C39—Pt2	111.6 (8)	C7—C6—C5	126.9 (9)
C7—C8—C9	118.6 (8)	C19—C18—C17	120.9 (12)
C7—C8—C17	120.2 (9)	C19—C18—H18	119.6
C9—C8—C17	121.3 (9)	C17—C18—H18	119.6
C26—C27—C28	120.6 (11)	N4—C28—C27	120.5 (8)
C26—C27—H27	119.7	N4—C28—C29	116.3 (9)
C28—C27—H27	119.7	C27—C28—C29	123.2 (10)
C22—C17—C18	116.6 (9)	C17—C22—C21	122.8 (12)
C22—C17—C8	122.1 (9)	C17—C22—H22	118.6
C18—C17—C8	121.3 (9)	C21—C22—H22	118.6
C45—C40—C41	116.5 (9)	C6—C7—C8	120.0 (9)
C45—C40—C31	121.9 (9)	C6—C7—H7	120.0
C41—C40—C31	121.6 (8)	C8—C7—H7	120.0
N3—C33—C32	120.5 (9)	N4—C24—C25	122.1 (11)
N3—C33—C34	112.5 (8)	N4—C24—H24	118.9
C32—C33—C34	127.0 (9)	C25—C24—H24	118.9
C8—C9—C10	120.6 (9)	C14—C13—C12	119.7 (11)
C8—C9—H9	119.7	C14—C13—H13	120.1
C10—C9—H9	119.7	C12—C13—H13	120.1
C40—C45—C44	121.7 (10)	C5—C4—C3	118.9 (10)
C40—C45—H45	119.1	C5—C4—H4	120.6
C44—C45—H45	119.1	C3—C4—H4	120.6
C32—C31—C30	117.8 (8)	C36—C35—C34	119.7 (11)
C32—C31—C40	121.2 (8)	C36—C35—H35	120.2
C30—C31—C40	120.9 (8)	C34—C35—H35	120.2
C42—C41—C40	121.4 (9)	N2—C5—C4	120.9 (9)
C42—C41—H41	119.3	N2—C5—C6	115.7 (8)
C40—C41—H41	119.3	C4—C5—C6	123.3 (9)
C11—C12—C13	118.3 (11)	C13—C14—C15	121.4 (10)
C11—C12—H12	120.9	C13—C14—H14	119.3
C13—C12—H12	120.9	C15—C14—H14	119.3
N1—C10—C9	118.8 (8)	C16—C15—C14	120.9 (10)
N1—C10—C11	112.1 (8)	C16—C15—H15	119.5
C9—C10—C11	129.1 (9)	C14—C15—H15	119.5
C41—C42—C43	122.0 (11)	C3—C2—C1	119.1 (11)
C41—C42—H42	119.0	C3—C2—H2	120.5
C43—C42—H42	119.0	C1—C2—H2	120.5
C42—C43—C44	117.5 (10)	N2—C1—C2	122.0 (11)
C42—C43—C46	122.1 (12)	N2—C1—H1	119.0
C44—C43—C46	120.4 (11)	C2—C1—H1	119.0
C24—N4—C28	118.4 (9)	C27—C26—C25	117.1 (11)
C24—N4—Pt2	129.8 (8)	C27—C26—H26	121.4
C28—N4—Pt2	111.6 (6)	C25—C26—H26	121.4
C33—C32—C31	120.0 (8)	C2—C3—C4	119.5 (11)
C33—C32—H32	120.0	C2—C3—H3	120.3
C31—C32—H32	120.0	C4—C3—H3	120.3
C30—C29—N3	119.2 (7)	C19—C20—C21	118.7 (10)
C30—C29—C28	128.0 (10)	C19—C20—C23	122.9 (13)
N3—C29—C28	112.8 (9)	C21—C20—C23	118.4 (12)
C24—C25—C26	121.1 (10)	C37—C36—C35	119.6 (11)
C24—C25—H25	119.4	C37—C36—H36	120.2
C26—C25—H25	119.4	C35—C36—H36	120.2
C1—N2—C5	119.6 (9)	C20—C23—H23A	109.5
C1—N2—Pt1	128.3 (7)	C20—C23—H23B	109.5
C5—N2—Pt1	112.1 (6)	H23A—C23—H23B	109.5
C15—C16—C11	115.5 (9)	C20—C23—H23C	109.5
C15—C16—Pt1	131.2 (8)	H23A—C23—H23C	109.5
C11—C16—Pt1	113.1 (7)	H23B—C23—H23C	109.5

Symmetry codes: (i) *x*+1, *y*, *z*.

### Hydrogen-bond geometry (Å, °)

**Table:**

*D*—H···*A*	*D*—H	H···*A*	*D*···*A*	*D*—H···*A*
C3—H3···Cg4^ii^	0.93	2.87	3.650 (14)	142
C14—H14···Cg5^iii^	0.93	2.71	3.445 (14)	136

Symmetry codes: (ii) −*x*+1, *y*+1/2, −*z*; (iii) −*x*, *y*−1/2, −*z*.
